# Comparative assessment of coronary physiology using transthoracic pulsed-wave Doppler and myocardial contrast echocardiography in rats

**DOI:** 10.1186/s41747-022-00319-4

**Published:** 2023-02-09

**Authors:** Sebastian Billig, Marc Hein, Mare Mechelinck, David Schumacher, Anna B. Roehl, Dieter Fuchs, Rafael Kramann, Moritz Uhlig

**Affiliations:** 1 Department of Anesthesiology, Faculty of Medicine, RWTH Aachen University, Pauwelsstraße 30, 52074 Aachen, Germany; 2 Institute of Experimental Medicine and Systems Biology, Faculty of Medicine, RWTH Aachen University, Pauwelsstraße 30, 52074 Aachen, Germany; 3 FUJIFILM VisualSonics, Inc., Joop Geesinkweg 140, 1114 AB Amsterdam, The Netherlands; 4 Division of Nephrology and Clinical Immunology, Faculty of Medicine, RWTH Aachen University, Pauwelsstraße 30, 52074 Aachen, Germany; 5 Department of Internal Medicine, Nephrology and Transplantation, Erasmus Medical, Doctor Molewaterplein 40, 3015 GD Rotterdam, The Netherlands

**Keywords:** Adenosine, Coronary vessels, Dobutamine, Echocardiography, Rats

## Abstract

**Background:**

Coronary physiology assessment in rodents by ultrasound is an excellent noninvasive and easy to perform technique, including pulsed-wave Doppler (PWD) and myocardial contrast echocardiography (MCE). Both techniques and the corresponding calculated parameters were investigated in this study at rest as well as their response to pharmacologically induced stress.

**Methods:**

Left ventricular myocardial function was assessed in eight anaesthetised rats using transthoracic echocardiography. Coronary physiology was assessed by both PWD of the left coronary artery and MCE using a bolus method. Measurements were performed at rest and under stimulation with adenosine and dobutamine. Effects of stimulation on the calculated parameters were evaluated and rated by effect size (*η*^2^).

**Results:**

Changes could be demonstrated by selected parameters of PWD and MCE. The clearest effect in PWD was found for diastolic peak velocity (*η*^2^ = 0.58). It increased from 528 ± 110 mm/s (mean ± standard deviation) at rest to 839 ± 342 mm/s (*p* = 0.001) with adenosine and 1093 ± 302 mm/s with dobutamine (*p* = 0.001). The most distinct effect from MCE was found for the normalised wash-in rate (*η*^2^ = 0.58). It increased from 1.95 ± 0.35% at rest to 3.87 ± 0.85% with adenosine (*p* = 0.001) and 3.72 ± 1.03% with dobutamine (*p* = 0.001).

**Conclusion:**

Induced changes in coronary physiology by adenosine and dobutamine could successfully be monitored using MCE and PWD in anaesthetised rats. Due to the low invasiveness of the measurements, this protocol could be used for longitudinal animal studies.

## Key points

- This study provides a protocol for pulsed-wave Doppler (PWD) and myocardial contrast echocardiography (MCE) in rats.
- Changes in physiology induced by pharmacological stress were assessed using PWD and MCE.
- Diastolic peak velocity and wash-in rate showed the greatest changes during stress.

## Background

Myocardial circulation is not only crucial in acute myocardial infarction [1] but also in the pathophysiology of ischemic cardiomyopathy [2] and aortic stenosis [3]. Therefore, the assessment of coronary physiology is of increasing relevance in different clinical and preclinical settings. In animal models, changes in coronary physiology are often a key feature to be assessed in response to a particular intervention or treatment [4, 5]. Measurements can be performed at the level of macro- (large coronary vessels) or microcirculation (myocardial tissue) and often require complex procedures, such as single-photon emission computed tomography, an invasive catheter or microsphere-based measurements [6–9].

Noninvasive techniques that are available even in small animals include single-photon emission computed tomography, magnetic resonance imaging, and echocardiographic techniques [9]. However, most of these techniques require a high level of expertise and the use of expensive, complex and rarely accessible equipment. In contrast echocardiography is much cheaper, less invasive, goes along with a shorter examination time and better simultaneous monitoring of the animal. It is also accessible to a much wider range of scientists. There are two common approaches to assess coronary physiology by transthoracic echocardiography: coronary artery blood flow can either be measured using pulsed-wave Doppler (PWD) [10, 11], or perfusion can be assessed using the flow kinetics of an intravenously applied contrast agent. The latter is referred to as myocardial contrast echocardiography (MCE) [1, 12, 13]. Although these techniques are readily available for small animals and a variety of parameters can be calculated, to the best of our knowledge, no work so far has applied both techniques and compared their response to pharmacological stress in one model.

Therefore, we designed an experimental study in anaesthetised rats to determine the most suitable parameters by their response to induced pharmacological stress with adenosine and dobutamine.

## Methods

In the present study, experiments were conducted on eight male Sprague Dawley rats (Charles River Laboratories International, Inc., Sulzfeld, Germany) with an average weight of 487 ± 20 g (mean ± standard deviation [SD]). The animals were housed in rat filter top cages (Type 2000, Tecniplast, Hohenpreisenberg, Germany) with a 12-h light-dark cycle under specific pathogen-free conditions in a temperature- and humidity-controlled environment (22 °C, 55% relative humidity). The animals were acclimatised for at least seven days before the start of the experiment. Throughout the trial, rats had ad libitum access to sterile, acidified water, and standard rat laboratory chow (Sniff GmbH, Soest, Germany). The experimental protocol was approved by the appropriate governmental institution (Landesamt für Natur, Umwelt und Verbraucherschutz NordrheinWestfalen, LANUV, Recklinghausen, Germany, Protocol No. 84–02.04.2016.A391). The animals in this study were part of an experimental design to investigate the influence of common bile duct ligation on myocardial function. Thus, four days before echocardiographic evaluation, the animals underwent a sham laparotomy without dissection of the common bile duct. Both, PWD and MCE were performed in all eight animals at rest and under pharmacologically induced stress as described below.

### Animal instrumentation

Adequate analgesia was ensured by subcutaneous injection of 10 μg/kg body weight buprenorphine (Temgesic, Essex Pharma GmbH – Msd Sharp & Dohme GmbH, Haar, Germany) 30 min before the procedure. Rats were then anaesthetised using 2 vol% isoflurane in 2 L/min oxygen. Spontaneous breathing via a nose cone was maintained throughout the experiment. Sufficient depth of anaesthesia was ensured by repeated testing of tail- and interdigital reflexes. Rats were fixed in a supine position on a feedback-loop controlled heating pad (TCAT-2LV controller, Physitemp, Clifton, USA). The thorax and ventral part of the neck were shaved. Next, a two-lumen central venous catheter (Multicath-2, 3Fr, Vygon, Ecquen, France) was inserted into the left external jugular vein using a cutdown technique.

### Echocardiography

**Fig. 1:**
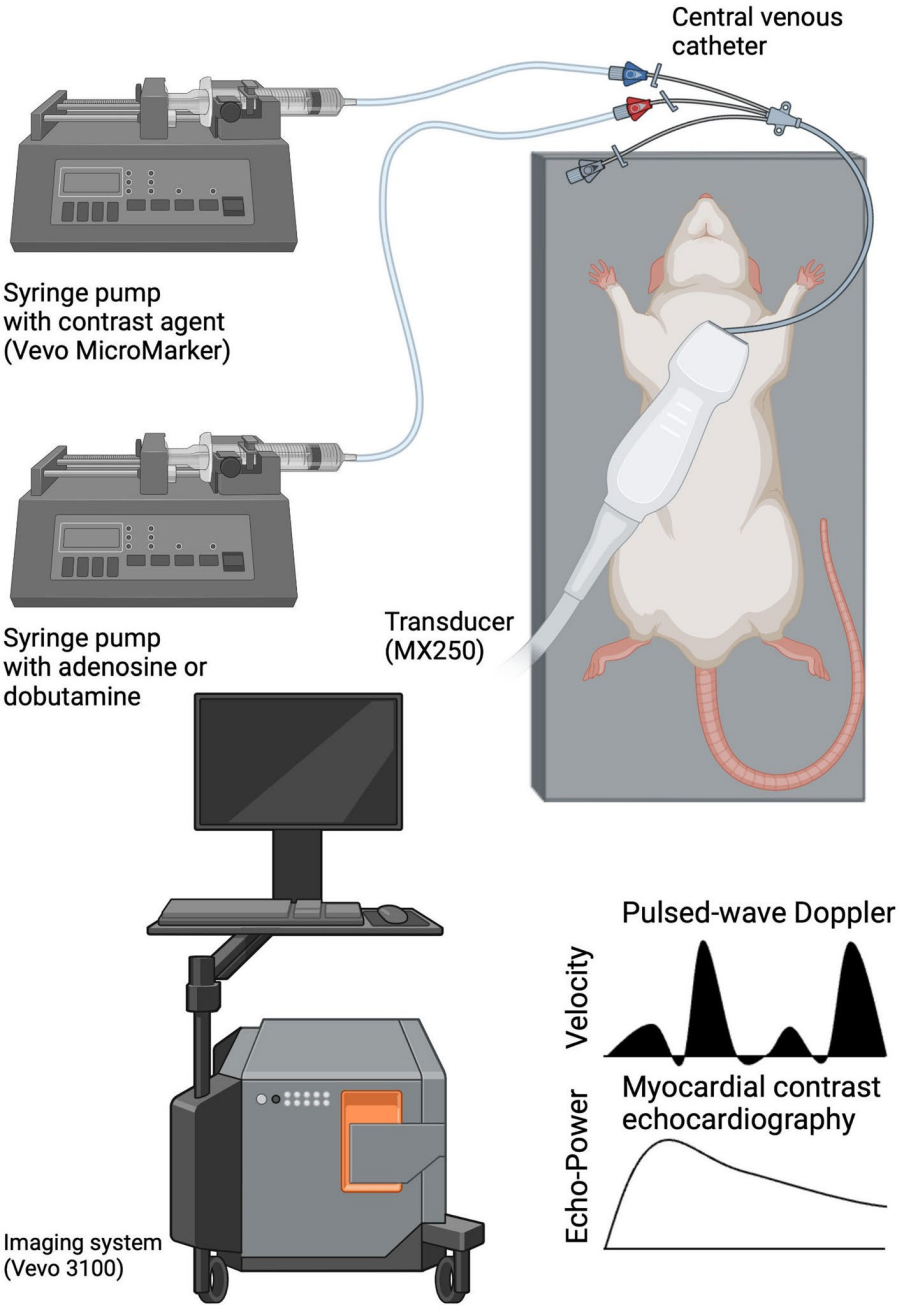
Schematic experimental setup with a rat in the supine position. Coronary circulation was studied by pulsed-wave Doppler of the left coronary artery and by myocardial contrast echocardiography. Measurements were performed under three conditions: baseline, adenosine, and dobutamine (created with Biorender.com)

**Fig. 2:**
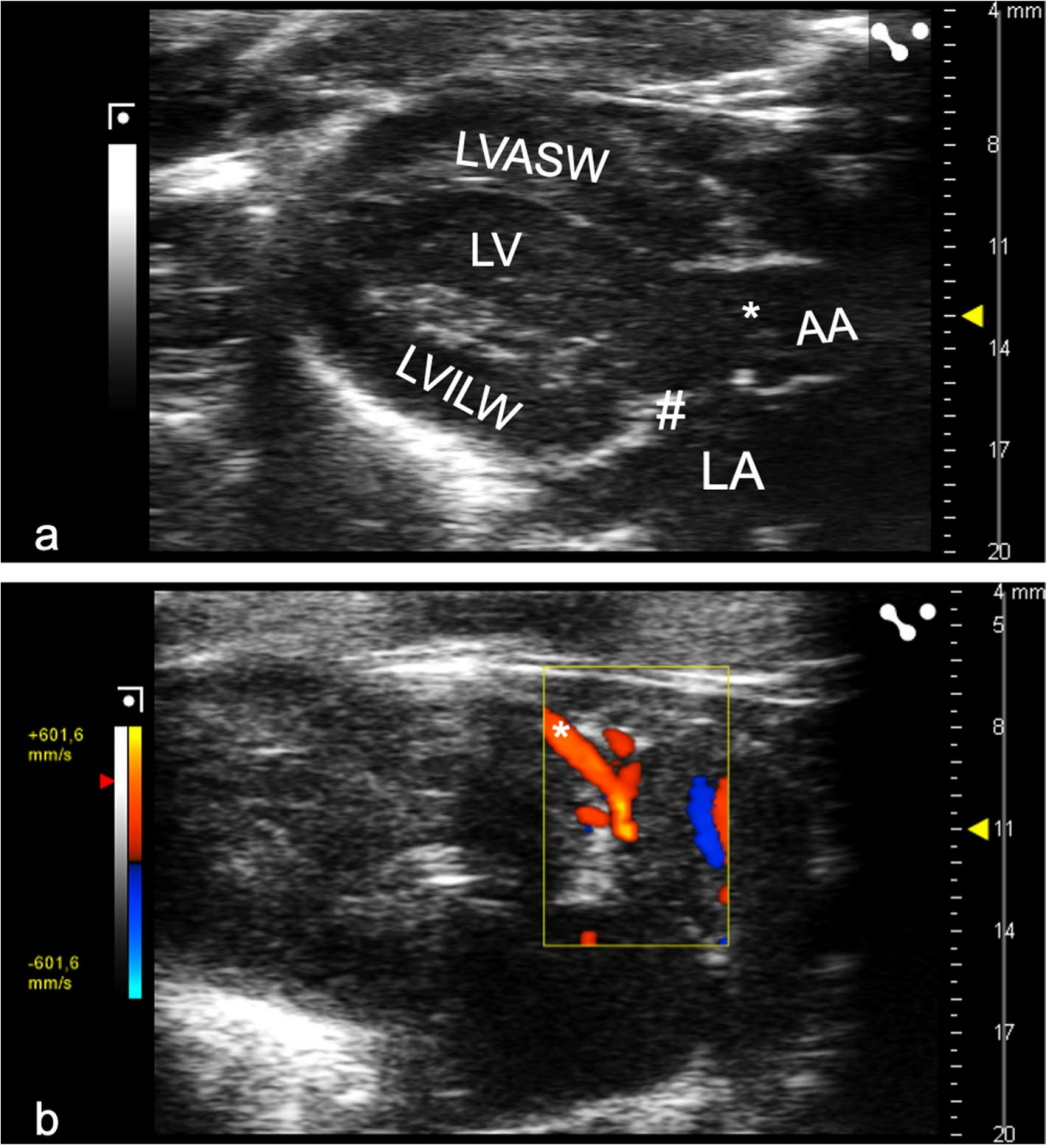
Representative B-mode and colour Doppler images acquired in a parasternal long axis (PLAX). **a** B-mode PLAX of the left ventricle. These images were used for volumetric and perfusion measurements. *AA* Ascending aorta, *LA* Left atrium, *LV* Left ventricle, *LVASW* Left ventricular anteroseptal wall, *LVILW* Left ventricular inferolateral wall, *aortic valve, *#* mitral valve. **b** Exemplary colour Doppler image of the proximal left coronary artery, which was assessed by pulsed-wave Doppler in the following step. *Proximal left coronary artery

Animals were transferred to a rat-handling platform (FUJIFILM Sonosite, Toronto, Canada) and received a continuous intravenous infusion of 10 mL/kg body weight/h Ringer’s solution to maintain a balanced volume status throughout the investigation period. Transthoracic echocardiography was conducted using a Vevo 3100 imaging system (FUJIFILM Sonosite, Toronto, Canada) equipped with an MX 250 linear array transducer (FUJIFILM Sonosite, Toronto, Canada) (Fig. 1). The transducer was attached to a stereotactic transducer mounting system (Vevo imaging station integrated rail system, FUJIFILM Sonosite, Toronto, Canada). Before images were recorded, animals were allowed to rest for 5 min to achieve a stable equilibrated hemodynamic state. A B-mode parasternal long axis (PLAX) of the left ventricle (Fig. 2a) was visualised, taking care to display the left ventricle at its maximum to avoid ventricular foreshortening. Three PLAX loops were recorded. All clips were stored with a frame rate of 100 frames/s and a length of 1 s for further offline analysis.

### Vital signs

ECG and noninvasive blood pressure were measured during the examinations. The 3-channel ECG was derived noninvasively via electrodes integrated into the rat-handling platform. The heart rate (HR) was determined from the ECG by identifying the QRS complexes. Noninvasive blood pressure was measured using an inflatable tail cuff with a pulse sensor (IN125/R, ADInstruments Ltd., Oxford, UK) that was connected to a data acquisition unit (PowerLab, ADInstruments, Colorado Springs, CO, USA). For data recording and analysis, Lab Chart (Lab Chart Pro 8.1, ADInstruments Ltd., Oxford, UK) was used. Systolic blood pressure (SBP) and diastolic blood pressure (DBP) were identified from the pulse signal.

### Pulsed-wave Doppler measurement

**Fig. 3:**
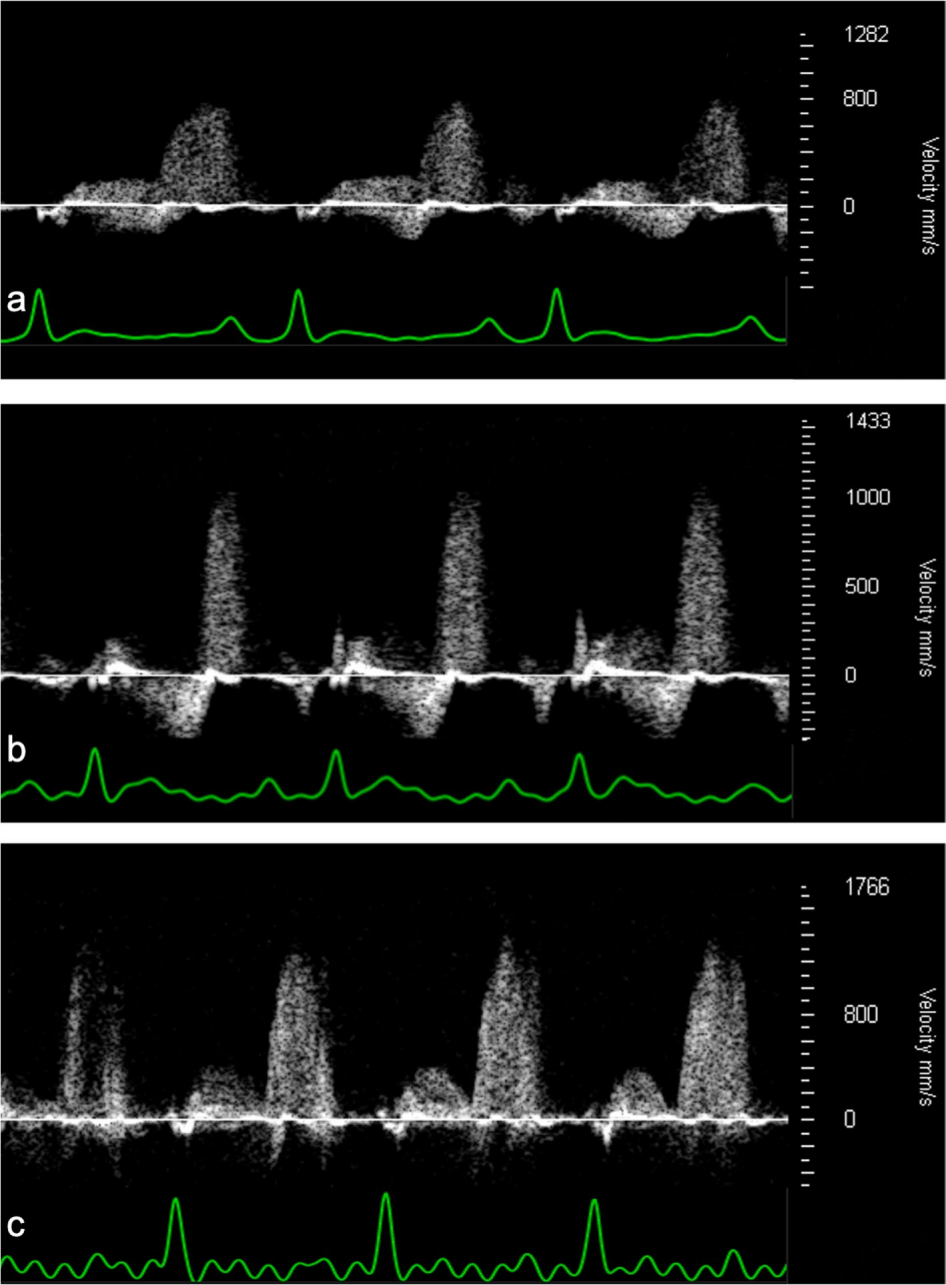
Representative pulsed-wave Doppler coronary flow patterns. The signals are shown with the corresponding electrodardiographic signal during baseline conditions (**a**), infusion of 140 μg/kg body weight/min adenosine (**b**), and infusion of 10 μg/kg body weight/min dobutamine (**c**)

The proximal segment of the left coronary artery was identified using Color Doppler in the PLAX. Small sideward adjustments in the transducer position allowed the left coronary artery to be visualised in a long axis in a modified PLAX (Fig. 2b). Next, PWD was applied in the proximal left coronary artery (Fig. 3). The angle correction was applied to optimise beam alignment with the blood flow.

### Myocardial contrast echocardiography measurement

**Fig. 4:**
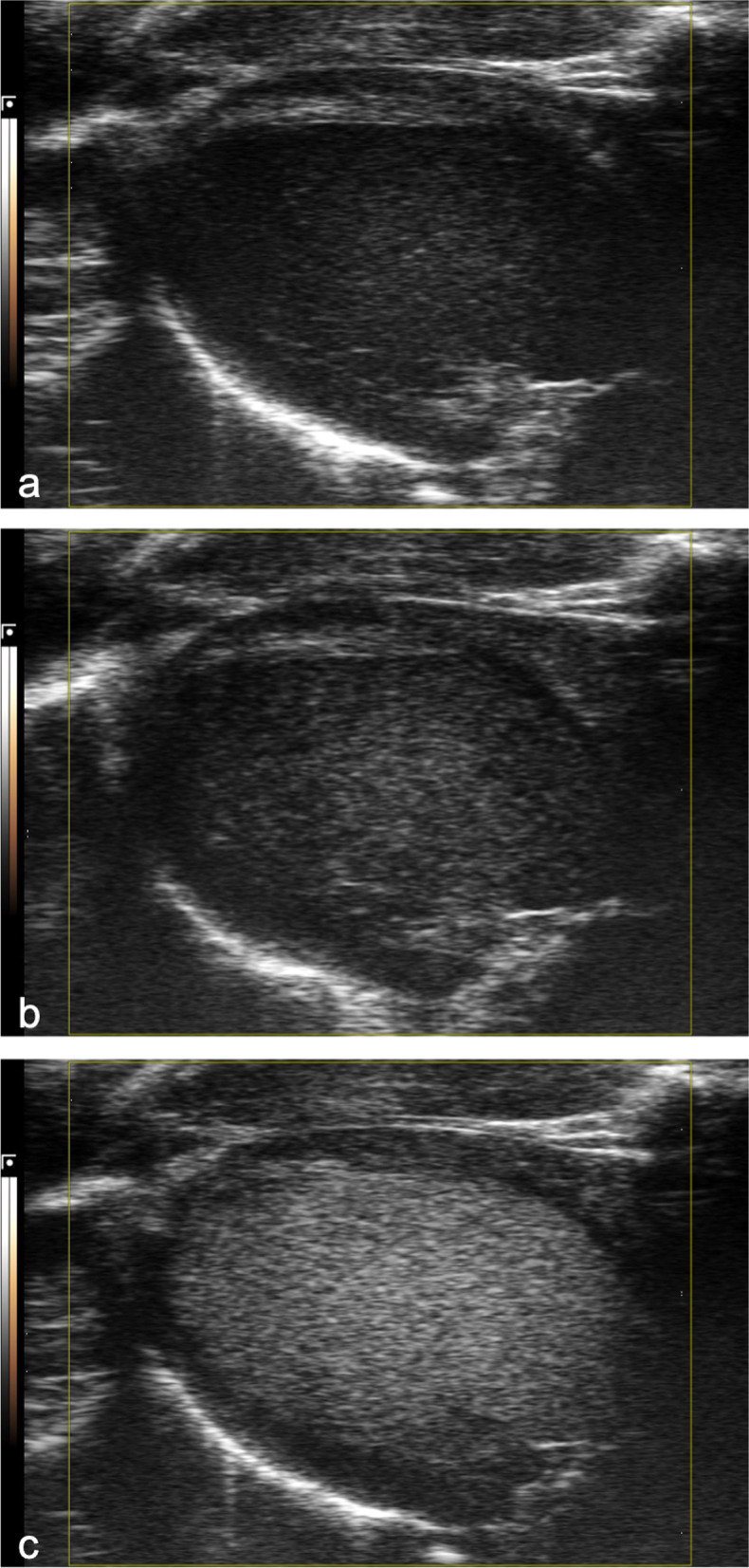
Representative time course of contrast activity in the lumen of the left ventricle. Shown are systolic gated modified parasternal long axis images that were acquired during nonlinear contrast imaging at the time of contrast agent arrival, *i.e.*, 0 s (**a**), 1 s (**b**), and 10 s (**c**) after contrast agent arrival

MCE was performed using the nonlinear contrast mode in a PLAX view of the left ventricle. Images were recorded ECG-gated, triggered by the R-wave in the ECG. A power of 10% and a dynamic range of 40 dB were used throughout the whole study in the nonlinear contrast mode. Vevo MicroMarker (FUJIFILM Sonosite, Toronto, Canada) was used as a contrast agent (phospholipid-coated perfluorobutane/nitrogen; median diameter 2.3–2.9 μm). Vevo MicroMarker was prepared by reconstituting the agent in 3.5-mL sterile saline. After slight agitation and a resting period of 10 min, the agent was diluted to a concentration of 2 x 10^8^ microbubbles/mL. The recording was activated, and a contrast agent bolus of 8 * 10^7^ microbubbles was injected intravenously over a period of 6 s using a syringe pump (Harvard Apparatus 11plus, Harvard Apparatus, Holliston, Massachusetts, US) (Fig. 4). This dilution and application period allowed for contrast measurements while the left ventricular anteroseptal wall and the inferolateral could be imaged. The recording was ceased after 60 s. B-mode imaging was activated to dissolve the contrast agent by ultrasound over 3 min. The MCE measurements were performed in triplicate.

### Stress echocardiography

The transthoracic echocardiography, Doppler, and MCE protocols described above were conducted at three stress levels (baseline condition, intravenous infusion of 140 μg/kg body weight/min adenosine, and intravenous infusion of 10 μg/kg body weight/min dobutamine). The images were acquired 5 min after each start of the continuous infusion to ensure the maximum effect of the drug. The measurement under dobutamine was performed at a 5-min interval from the last administration of adenosine, which corresponds to multiple half-life periods of adenosine [14]. At the end of the experiment, the animals were sacrificed under deep anaesthesia.

### Left ventricular function analysis

The stored cine loops were imported into Vevo Lab 5.6 (FUJIFILM Sonosite, Toronto, Canada) for offline analysis. The PLAX images were analysed by the build-in auto-LV function that offers an automated tracing of left ventricular endocardial borders. We obtained end-diastolic volume (EDV), end-systolic volume (ESV), stroke volume (SV), and cardiac output data from PLAX [15]. All shown data were averaged from three consecutive PLAX cine loops.

### Pulsed-wave Doppler analysis

The recorded Doppler loops were analysed using Vevo Lab 5.6. Coronary flow was assessed using the integrated automated envelope function in the PWD loops. Diastolic peak velocity (DPV) and systolic peak velocity (SPV) were defined as the maximum velocities during electrical diastole and systole. The mean velocity (MV) was obtained from the velocity time integral (VTI). The VTI was determined for the whole cardiac cycle (VTI) and separately for diastole (VTI_dia_) and systole (VTI_sys_). To account for the different heart rates caused by the stress test and thus normalise to 1 min, we multiplied the VTI by the HR. Furthermore, the ratio of systolic to diastolic blood flow velocity was calculated as a quotient of SPV and DPV. Coronary flow velocity reserve (CFVR) was determined for adenosine and dobutamine as the quotient of the DPV during pharmacological stress and the DPV at baseline [16]. All data shown were averaged from three consecutive cardiac cycles.

### Myocardial contrast echocardiography analysis

**Fig. 5:**
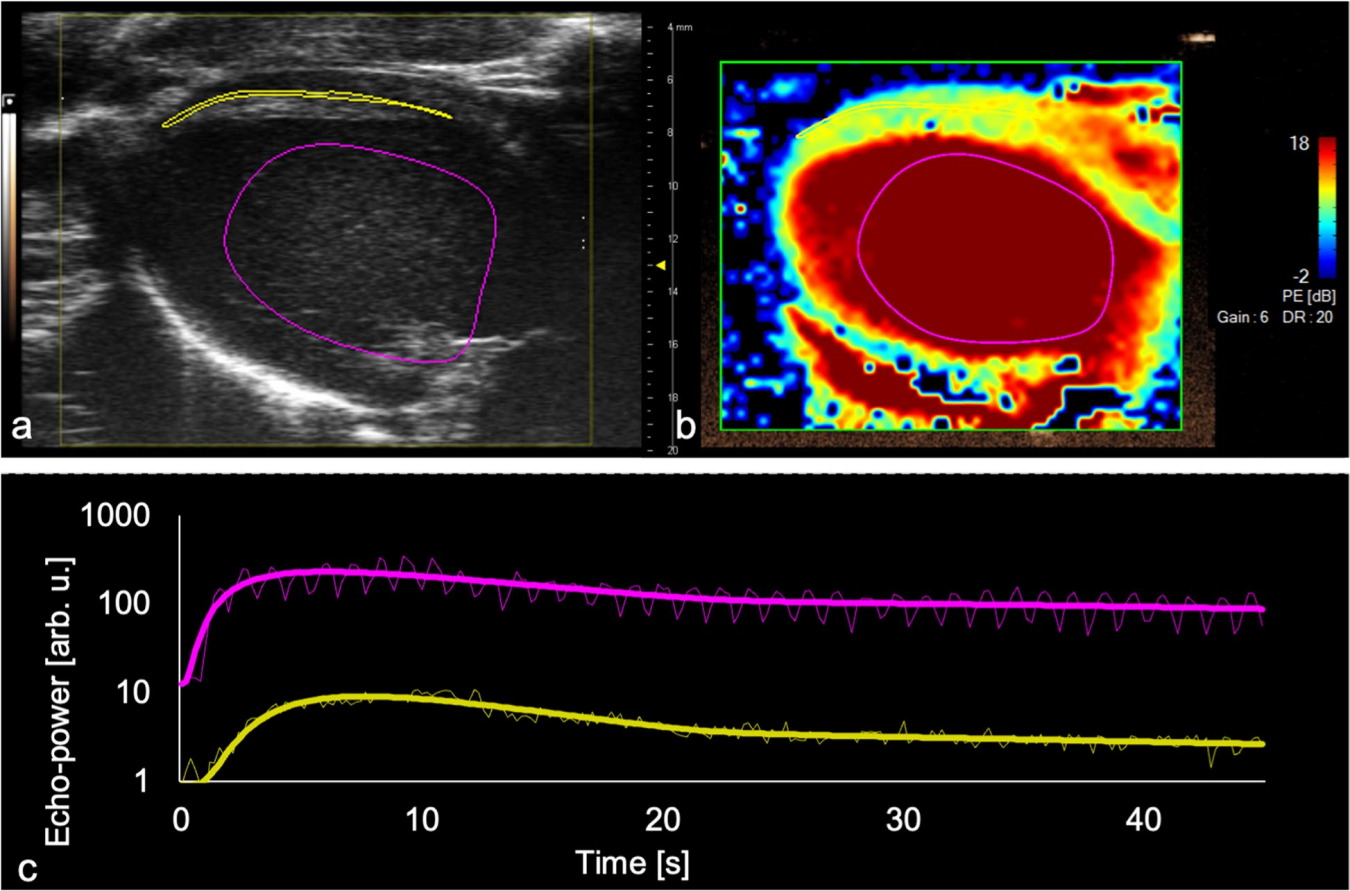
Illustration of the myocardial perfusion assessment in Vevo CQ. **a** Placement of the ventricular region of interest (ROI, pink) and the myocardial ROI (yellow). **b** Parametric colour-coded image showing the peak enhancement. **c** Time-intensity curve of the ventricular (pink) and myocardial (yellow) ROIs on a logarithmic scale. The bold line represents the fitted perfusion model, while the thin line represents the measured data points

MCE was analysed after importing the contrast loops into the Vevo CQ module (FUJIFILM Sonosite, Toronto, Canada). MCE loops were then trimmed to a length of 45 s after contrast agent arrival. A ventricular region of interest (ROI) was placed in the ventricular cavity, and a myocardial ROI was positioned in the anteroseptal wall of the left ventricle (Fig. 5a). For each ROI, Vevo CQ then calculated a fitting curve for a lognormal bolus model function for the signal power as a function of time (Fig. 5c). This curve was then used to calculate the corresponding perfusion parameters: peak enhancement (PE), which describes the maximum of the curve, wash-in rate (WIR), which represents the maximum slope of the wash-in curve and area under the curve (AUC). The obtained values were averaged between three consecutive bolus applications. For a reliable comparison between individuals, the data from the myocardial ROI were normalised to the data from the ventricular ROI for each animal and were then designated normalised values [1].

### Statistics

The statistical analysis was performed using SPSS (SPSS statistics Version 28, IBM, Armonk, NY, USA). The graphs were plotted using GraphPad PRISM 9 (GraphPad Software, San Diego, USA). Data are presented as the mean and the standard deviation. To analyse the difference between the different stress levels (baseline, adenosine, and dobutamine), a generalised linear model was fitted. The stress level was chosen as fixed effect, individual animals as subjects and values at different stress levels as repeated measures. Pairwise contrasts were added to enable a comparison between the individual stress levels. The effect size *η*^2^ was calculated to quantify the overall effect of the pharmacological stress on each measured parameter [17]. For that analysis, a confidence interval of 90% and the *F* values from the generalised linear model were used. A large *η*^2^ was defined as *η*^2^ > 0.14 [17]. To test for a correlation between the PWD and the MCE-derived parameters, a Pearson correlation test was performed. *p* values < 0.05 were considered statistically significant.

## Results

The procedures were well tolerated by all animals, and no adverse effects were observed. Three evaluable PLAX loops were recorded and analysed in each animal during baseline conditions as well as during adenosine and dobutamine infusion. In one animal, only one PLAX loop could be recorded and analysed due to technical issues. For MCE, we recorded three evaluable loops under each stimulation in every animal. SBP could not be evaluated in 9 of 72 measurements, and DBP could not be evaluated in 10 of 72 measurements due to insufficient signal quality.

### Hemodynamics and left ventricular function

**Fig. 6:**
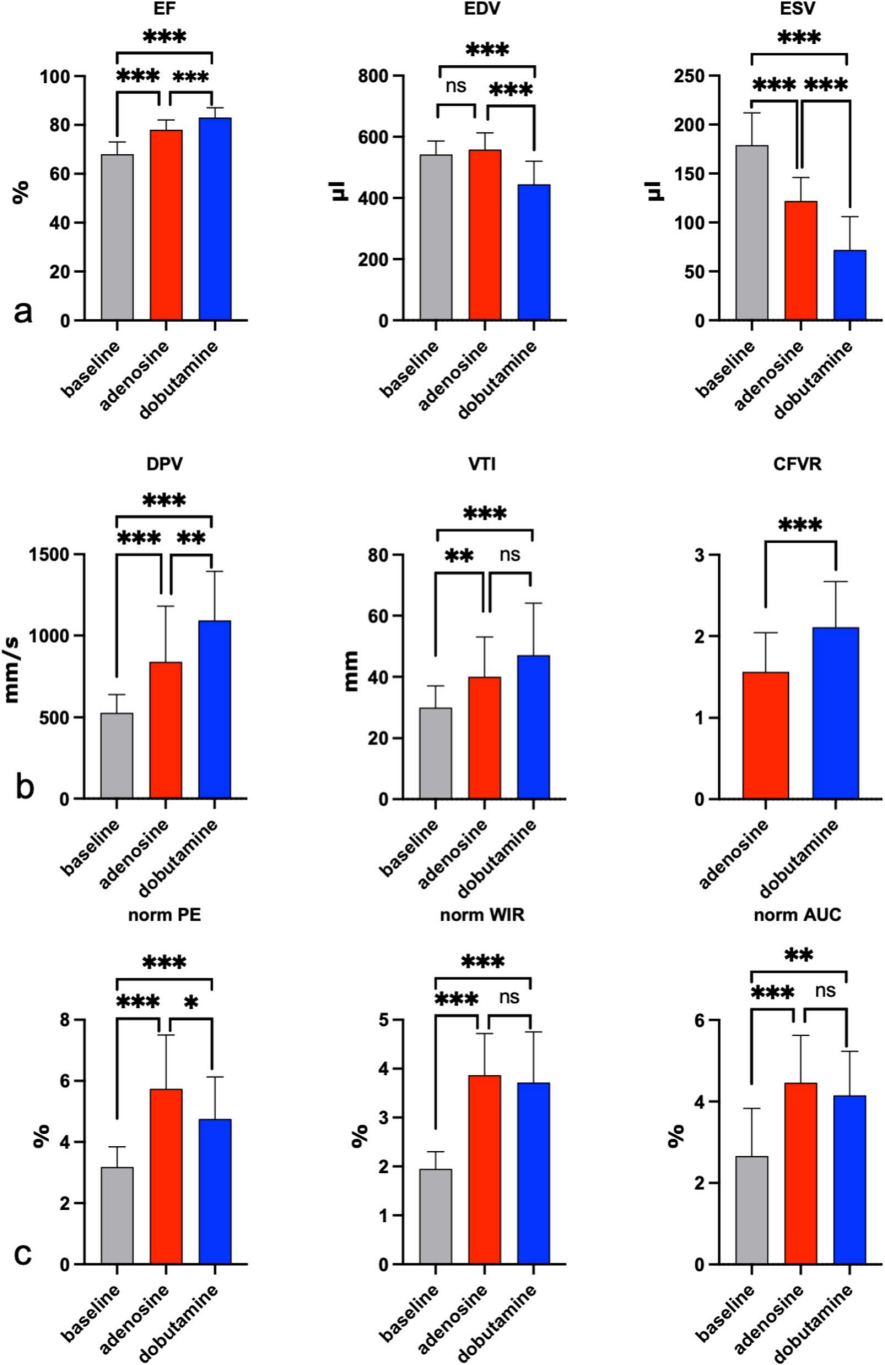
**a** Transthoracic echocardiography parameters from eight rats under baseline conditions and stimulation with adenosine and dobutamine: ejection fraction (EF), end-diastolic volume (EDV), and end-systolic volume (ESV). **b** Visualisation of the following pulsed wave Doppler-derived blood flow parameters in eight rats: diastolic peak velocity (DPV), velocity time integral (VTI), and coronary flow velocity reserve (CFVR). **c** Graphs showing the effect of adenosine and dobutamine on myocardial perfusion measured by myocardial contrast echocardiography (MCE) in eight rats using the following parameters: normalised peak enhancement (normalised PE), normalised wash-in rate (normalised WIR), and normalised area under the curve (normalised AUC). * *p* < 0.05; ** *p* < 0.01; ***p <0.001. Values are shown as the mean ± standard deviation

Table 1 shows the hemodynamic parameters, as well as the transthoracic echocardiography-derived parameters, for left ventricular myocardial function. Intravenous application of 140 μg/kg body weight/min adenosine induced a decrease in HR (344 ± 29 min^-1^ [mean ± SD] *versus* 320 ± 32 min^-1^, *p* = 0.007), SBP (116 ± 8 mmHg *versus* 92 ± 16 mmHg, *p* = 0.001), and DBP (58 ± 3 mmHg *versus* 48 ± 10 mmHg, *p* = 0.001). Pharmacological stress with adenosine did not show any alterations in cardiac output (130 ± 22 mL/min *versus* 139 ± 21 mL/min, *p* = 0.404). The EDV remained unaffected. However, adenosine led to a decreased ESV from 179 ± 33 μL to 122 ± 24 μL (*p* = 0.001), translating into an increase in SV (375 ± 41 μL *versus* 433 ± 40 μL, *p* = 0.001), and an increase in ejection fraction (EF) (68 ± 5% *versus* 78 ± 4%, *p* = 0.001) (Fig. 6a). Intravenous infusion of 10 μg/kg body weight/min dobutamine increased the HR to 405 ± 12 min^-1^ (*p* = 0.001). SBP showed no significant changes, while DBP showed a marginally significant decrease (*p* = 0.047). Dobutamine caused a reduction in EDV (542 ± 44 μL *versus* 445 ± 75 μL, *p* = 0.001) and in ESV (179 ± 33 μL *versus* 72 ± 34 μL, *p* = 0.001) (Fig. 6a). EF increased (68 ± 5% *versus* 83 ± 4%, *p* = 0.001) and cardiac output rose from 130 ± 22 mL/min to 150 ± 19 mL/min (*p* = 0.007).

**Table 1:** Hemodynamic and left ventricular parameters derived by transthoracic echocardiography at baseline and under stimulation with adenosine or dobutamine in eight rats

	Baseline		Adenosine			Dobutamine			*p value*^2^	*η*^2^
	Mean	SD	Mean	SD	*p value*^1^	Mean	SD	*p value*^1^		
HR (min^-1^)	344	29	320	32	0.007	405	12	0.001	0.001	0.73
SBP (mmHg)	116	8	92	16	0.001	112	13	0.298	0.001	0.36
DBP (mmHg)	58	3	48	10	0.001	54	6	0.047	0.001	0.35
EDV (μL)	542	44	558	55	0.315	445	75	0.001	0.001	0.37
ESV (μL)	179	33	122	24	0.001	72	34	0.001	0.001	0.61
SV (μL)	375	41	433	40	0.001	371	50	0.414	0.001	0.24
EF (%)	68	5	78	4	0.001	83	4	0.001	0.001	0.53
CO (mL/min)	130	22	139	21	0.404	150	19	0.007	0.075	0.11

*CO* Cardiac output, *DBP* Diastolic blood pressure, *EDV* End-diastolic volume, *EF* Ejection fraction, *ESV* End-systolic volume, *HR* Heart rate, *p value*^*1*^ Adenosine or dobutamine *versus* the baseline parameters, *p value*^*2*^ Adenosine *versus* dobutamine, *SBP* Systolic blood pressure, *SD* Standard deviation, *SV* Stroke volume, *η*^*2*^ Effect size

### Pulsed-wave Doppler

DPV, SPV, and MV increased during adenosine infusion (DPV: 528 ± 110 mm/s *versus* 839 ± 342 mm/s, *p* = 0.001; SPV: 169 ± 41 mm/s *versus* 265 ± 115 mm/s, *p* = 0.001; MV: 247 ± 55 mm/s *versus* 346 ± 129 mm/s, *p* = 0.001) (Fig. 6b, Table 2). The VTI and the separately analysed VTI_sys_, as well as the VTI_dia_, increased due to adenosine (VTI: 30 ± 7 mm *versus* 40 ± 13 mm, *p* = 0.001; VTI_sys_: 8 ± 3 mm *versus* 11 ± 5 mm, *p* = 0.002; VTI_dia_: 22 ± 6 mm *versus* 29 ± 10 mm, *p* = 0.011). DPV increased even more during dobutamine infusion than during adenosine infusion (DPV dobutamine: 1093 ± 302 mm/s, adenosine: 839 ± 342 mm/s, *p* = 0.005). Interestingly, SPV showed an increase under dobutamine that was at the same level as adenosine (SPV dobutamine: 274 ± 91 mm/s, adenosine: 265 ± 115 mm/s, *p* = 0.829). Accordingly, the SPV/DPV ratio was lower during dobutamine (0.26 ± 0.07) than under adenosine (0.33 ± 0.09) or baseline (0.33 ± 0.06). MV almost doubled from 247 ± 55 mm/s to 479 ± 165 mm/s (*p* = 0.001), which was significantly higher than MV during adenosine. The increased flow velocities resulted in a significantly higher VTI in the left coronary artery (30 ± 7 mm *versus* 47 ± 17 mm, *p* = 0.001). VTI and VTI_sys_ did not increase significantly under dobutamine compared to adenosine (VTI: *p* = 0.092; VTI_sys_: *p* = 0.33). CFVR was calculated to be 1.56 ± 0.48 for adenosine and 2.11 ± 0.56 for dobutamine.

**Table 2:** Pulsed-wave Doppler-derived blood flow values from the proximal left coronary artery of eight rats

	Baseline		Adenosine			Dobutamine			*p value*^2^	*η*^2^
	Mean	SD	Mean	SD	*p value*^1^	Mean	SD	*p value*^1^		
DPV (mm/s)	528	110	839	342	0.001	1093	302	0.001	0.005	0.58
SPV (mm/s)	169	41	265	115	0.001	274	91	0.001	0.829	0.32
MV (mm/s)	247	55	346	129	0.001	479	165	0.001	0.002	0.44
VTI (mm)	30	7	40	13	0.001	47	17	0.001	0.092	0.29
VTI_sys_ (mm)	8	3	11	5	0.002	10	3	0.008	0.330	0.17
VTI_dia_ (mm)	22	6	29	10	0.011	38	16	0.001	0.014	0.26
VTI * HR (mm/min)	10,342	2,647	12,899	4,935	0.004	19,058	6,691	0.001	0.002	0.37
SPV/DPV	0.33	0.06	0.33	0.09	0.814	0.26	0.07	0.003	0.003	0.16
CFVR			1.56	0.48		2.11	0.56		0.001	

*CFVR* Coronary flow velocity reserve, *DPV* Diastolic peak velocity, *MV* Mean velocity, *p value*^1^ Adenosine or dobutamine *versus* baseline parameters, *p value*^2^ Adenosine *versus* dobutamine, *SD* Standard deviation, *SPV* Systolic peak velocity, *VTI* Velocity time integral, *VTI*_*dia*_ Diastolic velocity time integral, *VTI*_*sys*_ Systolic velocity time integral, VTI*HR Velocity time integral * heart rate, *η*^*2*^ Effect size

### Myocardial contrast echocardiography

In the normalised values, adenosine led to an increase in the PE (3.18 ± 0.66 *versus* 5.74 ± 1.76%, *p* = 0.001), WIR (1.95 ± 0.35% *versus* 3.87 ± 0.85%, *p* = 0.001), and AUC (2.66 ± 1.17% *versus* 4.46 ± 1.16%, *p* = 0.001) of the anteroseptal myocardial wall (Fig. 6c, Table 3). During dobutamine infusion, the same effects were observed in the normalised values for PE, WIR, and AUC. Overall, the effect of adenosine on the normalised PE tended to be greater (normalised PE adenosine 5.74 ± 1.76% *versus* dobutamine: 4.76 ± 1.37%, *p* = 0.012). For the nonnormalised values, we observed a significant increase in the myocardial PE, WIR, and AUC during adenosine infusion compared to the baseline values. The *η*^2^ for these nonnormalised parameters was smaller than for the normalised values.

**Table 3:** Myocardial contrast echocardiography measurements in eight rats, each under baseline conditions, and under adenosine or dobutamine stimulation

	Baseline		Adenosine			Dobutamine			*p value*^2^	*η*^2^
	Mean	SD	Mean	SD	*p value*^1^	Mean	SD	*p value*^1^		
normalised PE (%)	3.18	0.66	5.74	1.76	0.001	4.76	1.37	0.001	0.012	0.44
normalised WIR (%)	1.95	0.35	3.87	0.85	0.001	3.72	1.03	0.001	0.269	0.58
normalised AUC (%)	2.66	1.17	4.46	1.16	0.001	4.15	1.08	0.003	0.143	0.20
Myocardium PE (a.u.)	7.04	4.30	11.2	6.80	0.019	8.54	5.03	0.423	0.081	0.08
Myocardium WIR (a.u.)	1.97	1.40	3.02	1.88	0.029	2.62	1.56	0.219	0.281	0.07
Myocardium AUC (a.u.)	228	167	384	261	0.010	290	182	0.573	0.029	0.09
Ventricle PE (a.u.)	225	122	193	101	0.131	189	113	0.871	0.779	0.05
Ventricle WIR (a.u.)	102	65.5	76	38.5	0.078	83	60.6	0.345	0.561	0.04
Ventricle AUC (a.u.)	10,820	7,727	8,887	6,081	0.193	8,095	5,985	0.084	0.601	0.04

*a.u.* Arbitrary unit, *AUC* Area under the curve, *p value*^1^ Adenosine or dobutamine *versus* baseline parameters; *p value*^2^ Adenosine *versus* dobutamine, *PE* Peak enhancement, *SD* Standard deviation, *WIR* Wash-in rate, *η*^*2*^ Effect size

### Correlations

**Fig. 7:**
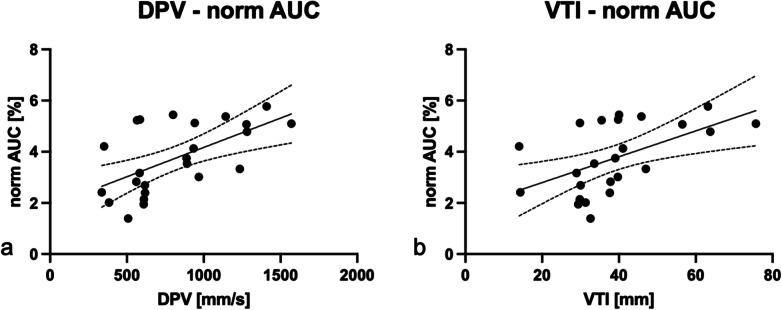
Correlation plots of pulsed-wave Doppler (PWD) and myocardial contrast echocardiography (MCE) parameters. **a** The diastolic peak velocity (DPV) derived from PWD showed a positive correlation with the normalised area under the curve (normalised AUC) derived from MCE (*r* = 0.59, *p* = 0.002). **b** The PWD-derived velocity time integral (VTI) showed a positive correlation with the normalised AUC (*r* = 0.54, *p* = 0.007) measured by MCE. Dotted lines represent 95% confidence interval

DPV showed a correlation with normalised WIR (*r* = 0.54, *p* = 0.007) (Fig. 7) and normalised AUC (*r* = 0.59, *p* = 0.002). The PWD-measured VTI showed a positive correlation with the MCE-derived values (VTI *versus* normalised PE: *r* = 0.45, *p* = 0.027; VTI *versus* normalised WIR: *r* = 0.46 *p* = 0.022; VTI *versus* normalised AUC: *r* = 0.54, *p* = 0.007) (Fig. 7).

## Discussion

With this study, we describe for the first time the effect of pharmacological stress on coronary physiology in a rat model by the MCE bolus method in comparison with PWD. A safe and simple protocol was established and suitable parameters with large changes and good reproducibility were identified: DPV for PWD and normalised WIR for MCE. Thus, changes in coronary physiology can be identified non-invasively. Adenosine and dobutamine are known to augment myocardial perfusion by different mechanisms. Coronary vasodilation during adenosine infusion is mediated by NO-dependent and NO-independent effects [18]. In rodents, the main effect is mediated by the NO-independent A_2_-adenosine receptor [19–21] that leads to relaxation of the coronary vascular smooth muscle cells [22] and causes a lowered resistance of the coronary vasculature. Dobutamine as a synthetic beta-agonist increases work and the resulting increased oxygen demand of the heart in turn is responsible for an increase in myocardial blood flow, which is further amplified by a direct vasodilator effect of dobutamine on the coronary vasculature [23, 24].

### Pulsed-wave Doppler

The recorded PWD signal of the left coronary artery showed a significant increase in coronary blood flow velocity during both adenosine and dobutamine infusion compared to baseline. The increase was detected in all measured velocities and the respective VTIs. Comparing our results with literature, we found a slightly higher peak velocity of 528 mm/s at baseline in our trial. Hägg et al. [25] measured a peak velocity of 440 mm/s in male Wistar rats anaesthetised with 1.6 vol% isoflurane, while Kelm et al. [16] reported 423 mm/s for female Fisher rats anaesthetised with 1.5–2 vol% isoflurane. Due to the lack of published coronary Doppler data under adenosine stress for rats thus far, we can only compare our data to other species. Wikstrom et al*.* [26] described a peak velocity of 640 mm/s for anaesthetised C57BL/6 mice with the same infusion rate of adenosine (140 μg/kg body weight/min adenosine). Interestingly, the peak velocity of 839 mm/s we observed during adenosine is comparable to a human coronary Doppler velocity of 790 mm/s determined in awake subjects with the same infusion rate [27]. Doppler data in rats for dobutamine stress echocardiography are also available to a very limited extent. Kelm et al*.* [16] reported a peak velocity of 1,005 mm/s under an infusion rate of 20 μg/kg body weight/min dobutamine, which is close to the value of 1,093 mm/s determined in our study. Meimoun et al. [27] reported a human peak velocity of 780 mm /s in awake patients. In addition to the peak flow velocity, we also addressed the flow velocities and VTIs separately for diastole and systole. These data are rarely mentioned in the literature; however, we think that they allow a more accurate assessment of the coronary blood circulation. For instance, the ratio of systolic to diastolic peak velocity or systolic to diastolic VTI was shifted towards a proportionally higher diastolic values during dobutamine infusion compared to baseline conditions or adenosine. A clinical study confirmed the larger effect of dobutamine on diastolic compared to systolic perfusion [28]; the difference was more pronounced in myocardial hypertrophy [28]. One of the most important Doppler-derived parameters is the CFVR since it can be used for the diagnosis and prognosis of coronary microvascular dysfunction [11, 16, 27, 29, 30]. Furthermore, CFVR can be used to compare the results between different experiments since it is a relative parameter. We observed a lower CFVR of 1.56 under adenosine treatment compared with 2.11 during dobutamine treatment. In line with our data, Wikstrom et al*.* [26] reported a CFVR of 1.73 for adenosine, and Kelm et al. [16] reported a CFVR of 2.1 for dobutamine.

### Myocardial contrast echocardiography

Using MCE, we were able to reliably detect an increase in myocardial perfusion in the analysed myocardial ROI under adenosine and dobutamine stress. During adenosine, the normalised values for PE as well as WIR roughly doubled compared to baseline. For a consistent contrast agent bolus, PE is indicative of the regional blood volume, while WIR is proportional to the regional blood flow [31]. Therefore, these results can be interpreted as an increase in the relative blood volume and flow. No comparable MCE studies under pharmacological stress have been published to date, especially not in a rat model. Troalen et al*.* [32] evaluated myocardial perfusion in isoflurane anaesthetised Wistar rats during 280 μg/kg body weight/min adenosine using magnetic resonance imaging, and they reported a 2.4-fold increase in myocardial blood flow. In another study using a positron emission tomography imaging technique, a 1.4-fold increase in blood flow was reported during 140 μg/kg body weight/min adenosine in isoflurane anaesthetised rats [33]. Although these results are only comparable to a limited extent with the results gained in our study due to differences in species, breeds, anaesthesia, and measurement techniques, they provide a rough reference. When comparing the observed effects under dobutamine stress to the literature, we found a slightly lower relative increase in WIR of approximately 1.9 compared with a 2.2-fold increase in myocardial blood flow, which was measured with positron emission tomography during an infusion of 20 μg/kg body weight/min dobutamine [33].

### Pulsed-wave Doppler *versus* myocardial contrast echocardiography

In general, it can be said that PWD is much easier to perform than MCE since no intravenous access must be established and the measurements are not only faster but also less invasive. Moreover, PWD does not require the use of a contrast agent. The use of the contrast agents in MCE is associated with high costs and possible damage to the myocardium, especially in destruction replenishment imaging. It may cause cardiomyocyte death or microvascular damage, although these effects are typically not observed at the low mechanical index that is used for MCE [34–36]. Due to the gating required for a precise localisation of tissue perfusion in the moving ventricle during MCE, either only diastole or systole can be analysed. In contrast, PWD also allows the separate measurement of systolic and diastolic flow velocities in a single step. Thus, observed effects can be separated by each phase of the cardiac cycle. A benefit of MCE compared to PWD is the possibility of capturing regional differences in perfusion. For example, Alvarez et al. [1] could show an impaired perfusion by MCE in LAD ligated mice in the corresponding myocardial segments. While both PWD and MCE measure different aspects in coronary physiology, we could show a moderate correlation between the two methods during stress echocardiography.

### Limitations

Isoflurane anaesthesia in rats has global hemodynamic effects and an isolated vasodilator effect on the coronary vasculature [8, 37]. However, this study could not be conducted without proper anaesthesia for ethical and practical reasons. We decided to use isoflurane because it is a widely used anaesthetic agent in rodent models and should therefore enable comparisons to further studies. Additionally, flow assessment in the LCA only consisted of a PWD in the left coronary artery without any measurements of the coronary vessel diameter. We could therefore only determine the blood flow velocity and VTI but not estimate the blood flow in the vessel. However, we decided against measuring an invasive reference because it would have interfered with the MCE measurements. Nevertheless, we chose this approach since it is a widespread simplification [25, 26, 38] that is also known to show fewer deviations than the integration of the lumen [26]. In addition, adenosine shows its vasodilator effect mainly on small vessels [39] and the vasodilator effect of dobutamine at the selected dosage on larger coronary vessels is also rather insignificant [23, 40]. Eventually, the order of dobutamine and adenosine was not randomised in our protocol, as an interaction seems to be highly unlikely due to the short half-life of adenosine.

In conclusion, appropriate parameters using MCE and PWD of the left coronary artery could be identified to display effects on macro- and microcirculation. Both methods yielded comparable results to the much more elaborate methods used in other studies (such as magnetic resonance imaging perfusion measurements or positron emission tomography). An advantage of transthoracic echocardiography-based methods is that simultaneously with coronary physiology ventricular function can be assessed with comparatively little effort. Due to the low invasiveness of the measurements, this protocol would allow serial measurements in a longitudinal study.

## Data Availability

The datasets generated and analysed in the present study are available from the corresponding author on reasonable request.

## Abbreviations

AUC: Area under the curve
CFVR: Coronary flow velocity reserve
DBP: Diastolic blood pressure
DPV: Diastolic peak velocity
EDV: End-diastolic volume
EF: Ejection fraction
ESV: End-systolic volume
HR: Heart rate
MCE: Myocardial contrast echocardiography
MV: Mean velocity
PE: Peak enhancement
PLAX: Parasternal long axis
PWD: Pulsed-wave Doppler
ROI: Region of interest
SBP: Systolic blood pressure
SD: Standard deviation
SPV: Systolic peak velocity
SV: Stroke volume
VTI: Velocity time integral
VTI_dia_: Diastolic velocity time integral
VTI_sys_: Systolic velocity time integral
WIR: Wash-in rate
